# Comprehensive Identification of the β-Amylase (BAM) Gene Family in Response to Cold Stress in White Clover

**DOI:** 10.3390/plants13020154

**Published:** 2024-01-05

**Authors:** Manman Li, Xiuhua Chen, Wangqi Huang, Kaiyue Wu, Yan Bai, Donglin Guo, Changhong Guo, Yongjun Shu

**Affiliations:** 1 Key Laboratory of Molecular Cytogenetics and Genetic Breeding of Heilongjiang Province, College of Life Science and Technology, Harbin Normal University, Harbin 150025, China; limanman1208@stu.hrbnu.edu.cn (M.L.); guodonglin@hrbnu.edu.cn (D.G.); kaku3008@hrbnu.edu.cn (C.G.); 2 International Agriculture Research Institute, Yunnan Academy of Agricultural Sciences, Kunming 650200, China; cxh@yaas.org.cn; 3 National Engineering Research Center for Ornamental Horticulture, Yunnan Flower Breeding Key Laboratory, Flower Research Institute, Yunnan Academy of Agricultural Sciences, Kunming 650200, China; hwq@yaas.org.cn

**Keywords:** white clover, BAM, cold stress, genetic regulation network, qRT-PCR

## Abstract

White clover (*Trifolium repens* L.) is an allopolyploid plant and an excellent perennial legume forage. However, white clover is subjected to various stresses during its growth, with cold stress being one of the major limiting factors affecting its growth and development. Beta-amylase (BAM) is an important starch-hydrolyzing enzyme that plays a significant role in starch degradation and responses to environmental stress. In this study, 21 members of the BAM gene family were identified in the white clover genome. A phylogenetic analysis using *BAMs* from Arabidopsis divided *TrBAMs* into four groups based on sequence similarity. Through analysis of conserved motifs, gene duplication, synteny analysis, and *cis*-acting elements, a deeper understanding of the structure and evolution of *TrBAMs* in white clover was gained. Additionally, a gene regulatory network (GRN) containing *TrBAMs* was constructed; gene ontology (GO) annotation analysis revealed close interactions between *TrBAMs* and AMY (α-amylase) and DPE (4-alpha-glucanotransferase). To determine the function of *TrBAMs* under various tissues and stresses, RNA-seq datasets were analyzed, showing that most *TrBAMs* were significantly upregulated in response to biotic and abiotic stresses and the highest expression in leaves. These results were validated through qRT-PCR experiments, indicating their involvement in multiple gene regulatory pathways responding to cold stress. This study provides new insights into the structure, evolution, and function of the white clover BAM gene family, laying the foundation for further exploration of the functional mechanisms through which *TrBAMs* respond to cold stress.

## 1. Introduction

The environment in which a plant grows is a major factor in its growth. When there are drastic changes in factors, such as water availability, light exposure, soil conditions, nutrients, temperature, humidity, air quality, and the presence of microorganisms as well as animals in the surrounding environment, impacting the normal growth and development of plants, this phenomenon is referred to as plant stress or adversity [1]. Temperature is one of the significant factors affecting geographical distribution, growing seasons, and the yield of crops; cold stress can have a severe impact on the growth and development of crops [2,3]. The main effects of cold stress on plants primarily manifest in terms of membrane systems, enzyme activities, cell dehydration, and disruptions in cellular metabolism. Under cold stress, cells experience osmotic stress, which can lead to imbalanced turgor pressure, disrupting the integrity of cell membranes, affecting plant metabolism, slowing down the plant’s growth and development, and, in severe cases, leading to plant mortality [4,5]. Therefore, enhancing a plant’s cold resistance is an effective way to reduce the adverse effects of cold stress, increase overwintering ability, and improve a plant’s capacity to withstand cold stress, thus serving as an effective approach for plants to defend against cold stress conditions [6].

The rapid accumulation of non-structural carbohydrates is a crucial initial metabolic response of plants to cold stress [7]. Plants store carbohydrates, such as starch and fructans, through photosynthesis as storage materials that can be utilized to fulfill their energy requirements. Starch, composed of glucose polymers arranged in osmotically inactive granules, not only serves as a significant energy source but also plays a role in enhancing the frost resistance of plants as a non-structural carbohydrate [5,8]. In the leaves of green plants, starch accumulates in chloroplasts during the day and undergoes degradation at night when photosynthesis is inactive, thereby supplying sugars to the tissues [9]. Starch degradation primarily involves the five enzymes of α-amylase (AMY), β-amylase (BAM), limit dextrinase (PUL), β-glucosidase, and α-glucan phosphorylase (PHO). Among these, BAM is a fundamental enzyme responsible for starch degradation. Functioning as an exo-acting enzyme, BAM sequentially cleaves α-1,4-glycosidic linkages from the non-reducing ends of polysaccharides, liberating maltose units [10,11,12].

BAM is a crucial type of starch-hydrolyzing enzyme that plays a significant role in starch degradation, especially when exposed to environmental stress. BAM belongs to the typical exo-acting hydrolytic enzymes and its primary function is to break down starch in plants, yielding maltose as a product [13]. BAM is a member of glycoside hydrolase family 14 (Glyco_hydro_14) [14]. All known BAM proteins contain three sequence regions that are highly conserved. With the N-terminal region of BAM, there exists a characteristic glycoside hydrolase domain (PF01373) that encompasses aspartic acid residues implicated in catalytic activity. The second domain is located at the center of the protein’s three-dimensional spatial structure, with glutamic acid at its core, which also plays a role in the catalytic mechanism [13,15]. Furthermore, the crystal structure of the BAM-maltose complex reveals that active site residues Glu186 and Glu380 are strictly conserved in BAM proteins [16]. *Arabidopsis thaliana* contains nine *BAM* genes, but only four of them have enzymatic catalytic activity [8]. *AtBAM1* encodes a cytoplasmic BAM with catalytic activity in leaves and it is the main gene responsible for starch degradation in leaf cells in response to drought or salt stress [17]. Kaplan et al. reported that *AtBAM1* accounts for over 90% of the total BAM activity in Arabidopsis leaf cells [18]. Additionally, *AtBAM1* helps regulate stomatal opening and osmotic balance. Li et al. reported that BAM activity encoded by *AtBAM2* is much lower than that of *AtBAM1* or *AtBAM3* due to the insertion of four amino acids into the surface loop near the active site [19]. Furthermore, after cold treatment, the transcript abundance of *AtBAM3* and the content of reducing sugars increase in the *bam5/1* Arabidopsis mutant [8]. *BAM3* encodes a plastid-localized enzyme with catalytic activity expressed in leaf cells, playing an essential role in nighttime leaf starch degradation [18,20]. The potato (*Solanum tuberosum*) homologue of *BAM3* exhibits similar functionality [21].

Light, low temperature, drought, high salinity, and osmotic stress can induce the expression and protein activity of *BAM* genes [22,23]. It has been observed that *BAM* transcription is activated during temperature stress, leading to an increase in maltose content [24]. Monroe et al. reported that *BAM3* contributes to chloroplast starch degradation in leaf cells during nighttime and cold stress [8]. Overexpression of *PtrBAM1* in tobacco leads to increased BAM activity and starch degradation, causing the accumulation of maltose and soluble sugars at room temperature or under cold stress conditions [24]. The role of BAM in cold tolerance has been observed in many other plants. Cold stress can induce the expression of *PrbBAM3* in birch (*Pyrus betulaefolia*), which is inhibited through maltose [25]. Additionally, overexpression of *PbrBAM3* in *Nicotiana benthamiana* and pear increased BAM activity promoted starch degradation under cold treatment and enhanced plant cold tolerance [25]. Overexpression of *AbBAM3.1* induced through cold stress enhances the freezing resistance of transgenic plants in kiwifruit (Actinidia arguta) and Arabidopsis [26]. Suppression of *StBAM1* expression results in low BAM activity and reduced cold resistance in potato plants [27].

White clover (*Trifolium repens* L.) is an excellent perennial legume forage, characterized as an allopolyploid species originating from Southeastern Europe and South Asia. It is rich in various nutrients and mineral elements, possessing high nutritional, ecological, genetic breeding, and medicinal values [28,29]. This forage also has excellent palatability, with high carbohydrate and protein content, making it widely used as ruminant animal feed in many parts of the world [30]. However, white clover inevitably faces various abiotic stresses during its growth, such as high salinity stress, drought stress, and cold stress, especially in high-latitude regions where severe winters can lead to abnormal death in white clover, significantly impacting its production and promotion [31]. Consequently, enhancing white clover’s cold tolerance has become a critical research focus. Compared with related species like alfalfa and soybean, the structural and genetic information for white clover has been limited, particularly at the genome level, which significantly restricts its breeding and improvement [32,33,34]. It was not until 2019 that the white clover genome sequence was made available, resolving the genetic background of white clover [35]. Researchers are now able to study gene functions at the genome level, contributing significantly to genetic improvement in white clover. For instance, Li et al. identified 145 *WRKY* genes in the white clover genome and identified some *TrWRKY* genes that can actively respond to cold stress [36].

In this study, bioinformatic methods were employed to identify the members of the BAM gene family from the entire white clover genome. Their phylogenetic relationships, conserved motifs, *cis*-acting elements, chromosomal distribution, and genetic regulatory networks were analyzed. Additionally, the expression profiles of *TrBAM* genes under various tissues and stresses were investigated using RNA-seq and qRT-PCR. This work enhances our understanding of the evolutionary patterns of the TrBAM gene family, laying a solid foundation for further research into the functional responses of *TrBAM* genes to cold stress.

## 2. Results

### 2.1. Identification of BAM Genes in White Clover

The HMMER and Pfam numbers (PF01373) were utilized to conduct a search for BAM protein sequences in the white clover genome database, resulting in the identification of white clover *BAM* genes. An expect (e) cutoff of 0.01 was applied to eliminate redundant sequences. In total, 21 TrBAM protein sequences were successfully identified. These genes were designated as *TrBAM1*–*TrBAM21* based on their homologous sequences in Arabidopsis genes (Table 1). Comprehensive genomic information for these *TrBAM*s, including their names, gene loci, chromosomal positions, intron numbers, and protein lengths (in amino acid residues), was compiled and is presented in Table 1. Of these genes, *TrBAM16* and *TrBAM17* encoded the longest proteins, encompassing 700 amino acid residues, while *TrBAM15* encoded the shortest protein, with only 171 amino acid residues. It is noteworthy that the *TrBAMs* contained introns, with the number of introns varying between two and nine. Among the 21 *TrBAMs*, eight genes exhibited homology to *AtBAM3*. The distribution of these *TrBAMs* across the 16 chromosomes of white clover was uneven, with a predominant presence on chromosomes one, two, four, five, six, seven, and eight. The result of subcellular localization prediction showed that most TrBAMs were located in the nucleus; the others were predicted to exit in the chloroplast, or cytosol (Table S1).

### 2.2. Phylogenetic Analysis of the BAM Gene Family among Arabidopsis and White Clover

**Figure 1:**
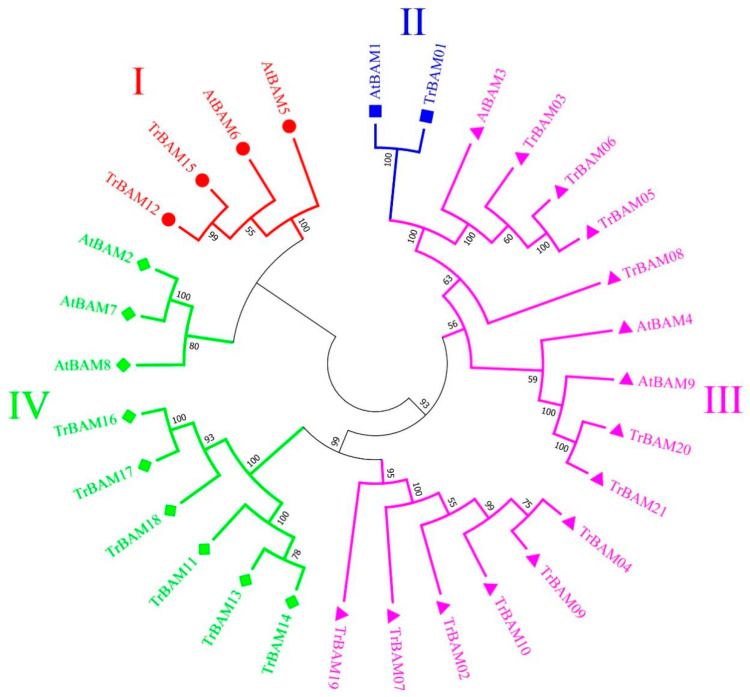
Phylogenetic analysis of white clover BAM proteins. The NJ tree was constructed from the amino acid sequences of TrBAM using MEGA11 with 1000 bootstrap replicates. The white clover BAM proteins were grouped into four groups (Group I labeled as red solid circles, Group II labeled as blue solid squares, Group III labeled as pink solid triangles, and Group IV labeled as green solid diamonds). The number at the node was the bootstrap value, representing the confidence level of the branch.

An unrooted neighbor-joining (NJ) tree was constructed to illustrate the phylogenetic relationships among TrBAMs (Figure 1). The 21 TrBAMs were categorized into four primary subfamilies based on the tree’s topology aligning with the classification of AtBAMs (Figure 1). In Figure 1, Group III exhibited the largest number of proteins, comprising 11 BAM proteins from white clover and three from Arabidopsis. Group I encompassed five BAM proteins from white clover and two from Arabidopsis. Group IV included four BAM proteins from white clover and three from Arabidopsis. Group II was the most modest, featuring a solitary BAM protein from both white clover and Arabidopsis. In addition, the classification bootstrap values of Group I, II, and IV reached 80%, indicating that the classification results are credible. The molecular phylogenetic tree vividly demonstrated a remarkable consistency in the distribution of BAMs between white clover and Arabidopsis.

### 2.3. Motif Composition Distribution Analysis of TrBAM Proteins in White Clover

**Figure 2:**
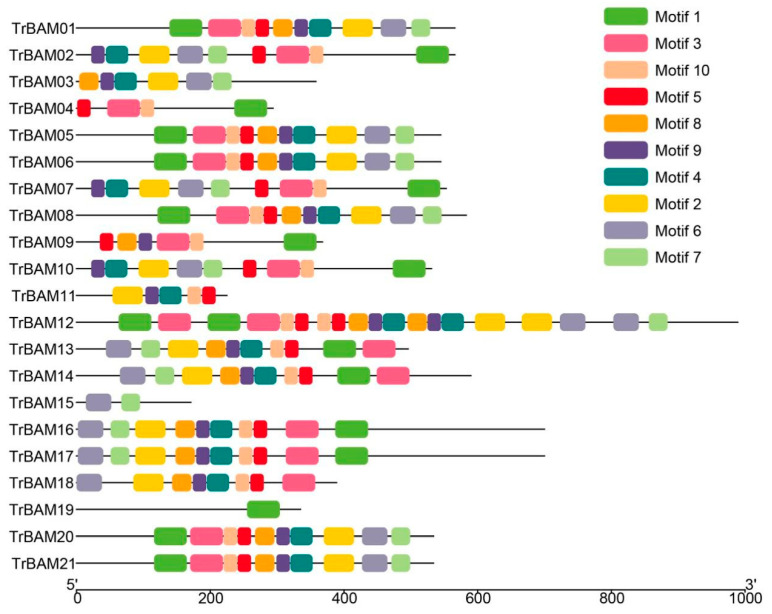
Distribution of conserved motifs of TrBAMs in white clover.

The MEME software (version 4.8.1) was employed to perform a rigorous analysis of the amino acid sequences of BAM, leading to the discovery of 10 conserved motifs (Figure 2). Remarkably, these 10 conserved motifs were identified across groups I, II, III, and IV. As illustrated in Figure 2, the TrBAMs in Group III exhibited the presence of all 10 motifs, with the majority containing motifs one, four, and six. Motif one was found to contain “Glyco_hydro_14” residues (Figure S1), confirming the existence of the “Glyco_hydro_14” domain in these TrBAMs. Motifs four and six were detected in TrBAMs that lacked motif one, yet they also harbored “Glyco_hydro_14” residues (Figure S1). These findings indicated a divergence in the “Glyco_hydro_14” domain within white clover. In addition, motif two and motif three also contained the “Glyco_hydro_14” residues. Groups I, II, and III exhibited analogous results, with each TrBAM featuring motif one, motif four, or motif six as well as, in some instances, all three motifs, in agreement with the BLAST and domain search results. The proteins in each group exhibited a comparable quantity and variety of motifs, indicating functional resemblances among these TrBAMs.

### 2.4. Cis-Acting Element Analysis of the TrBAM Promoter

**Figure 3:**
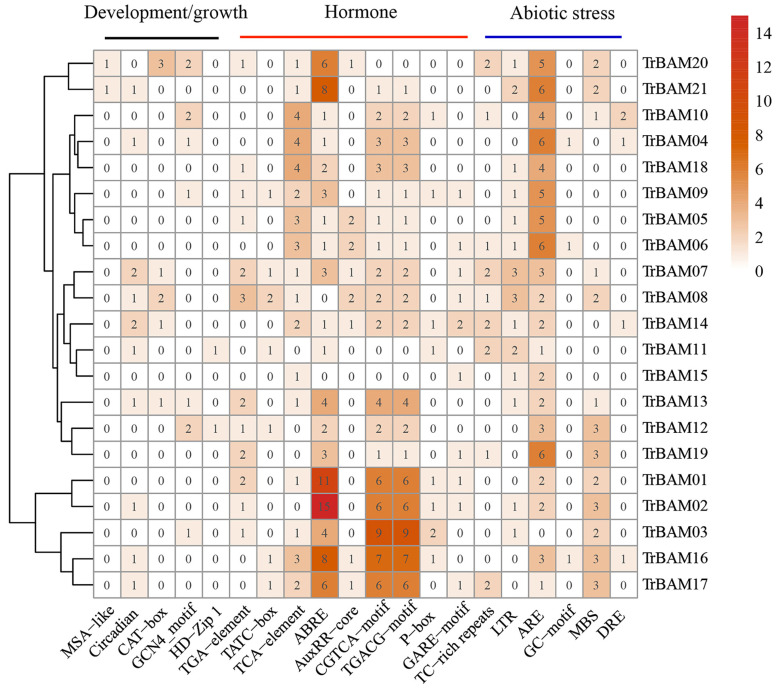
Three types of *cis*-acting elements were identified in the promoter regions of *TrBAMs*. Darker colors indicate higher numbers of *cis*-acting elements.

To gain deeper insights into the potential functions and regulatory mechanisms of *TrBAM* genes, we conducted an analysis of *cis*-acting elements within their promoter sequences using PlantCARE online tool (http://bioinformatics.psb.ugent.be/webtools/plantcare/html/ (accessed on 15 September 2023)) (Figure 3). Our analysis revealed three main categories of *cis*-acting that were present in the 2 kb promoter regions of the *TrBAMs* as follows: those related to growth and development, hormones, and stress responses. Within the category of growth and development elements, we identified a cell cycle regulatory element (MSA-like, TCCAACGGT), circadian rhythm regulatory element (circadian, CAAAGATATC), meristematic tissue expression element (CAT-box, GCCACT), endosperm expression element (GCN4-motif, TGAGTCA) and fenestrated chloroplast differentiation element (HD-Zip1, CAAT(A/T)ATTG). Within the category of hormone elements, we identified that hormone-related elements included a abscisic acid-related element (ABRE, ACGTG), gibberellin-related element (GARE-motif), salicylic acid-related element (TCA-element, CCATCTTTTT and TCAGAAGAGG), and methyl jasmonate (MeJA)-related elements (CGTCA-motif and TGACG-motif). Within the category of stress-responsive elements, in terms of abiotic stress responses, we observed the presence of the hypoxia-specific-inducible element (GC-motif, CCCCCCG), anaerobic-inducible element (ARE, AAACCA), drought-inducible cis-element (MBS, CAACTG), low-temperature-responsive cis-element (LTR, CCGAAA), and dehydration-responsive cis-elements (DRE, GCCGAC and TCGAC). A noteworthy observation is the significant occurrence of multiple ABRE motifs in a substantial proportion of *TrBAM*s, suggesting their potential for responding to abscisic acid under stress conditions. Furthermore, the promoter region of 20 *TrBAMs* contained ARE elements essential for anaerobic induction, indicating a potential role in anaerobic stress responses. Additionally, we identified low-temperature responsive elements (LTR), drought-inducibility elements (MBS), and TC-rich repeats in the promoter regions of 14, 13, and nine *TrBAMs*, respectively. This implies that these genes may play a crucial role in responding to various abiotic stresses in white clover. Moreover, DRE binding sites specifically recognized by *CBF* genes were found in the promoter regions of some genes, including *TrBAM04*, *TrBAM10*, *TrBAM14*, and *TrBAM16*.

### 2.5. Chromosome Localization, Gene Duplication and Ka/Ks Analysis of TrBAM Genes in White Clover

**Figure 4:**
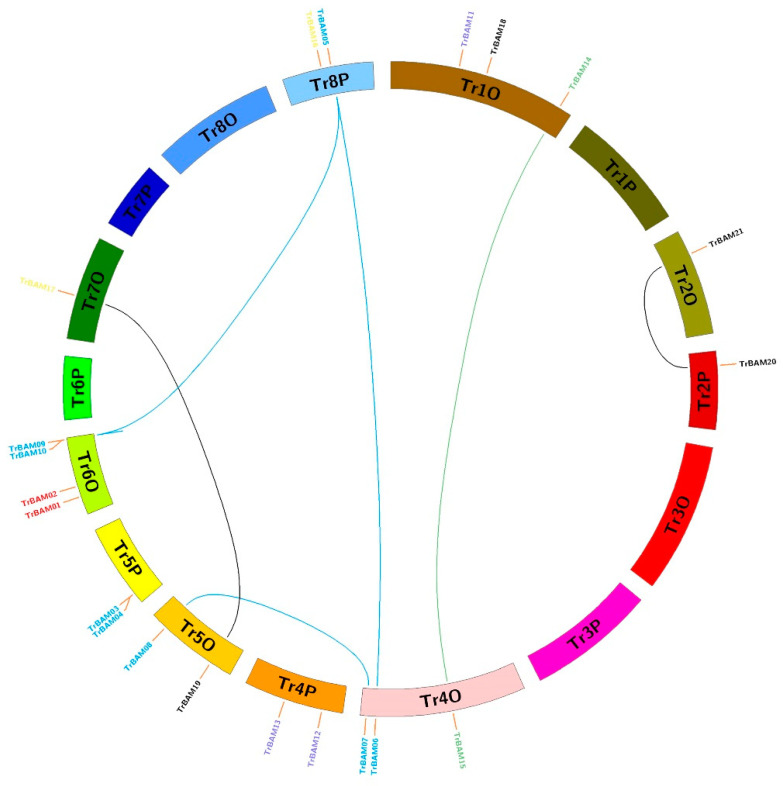
Chromosome distribution and expansion analysis of *BAMs* in white clover. Green lines show duplications between members of Group I; blue lines show duplications between members of Group III; greys lines show duplications between members of Group IV.

To explore the evolutionary dynamics and expansion of *BAMs* in white clover, we employed MCScanX (version python) and Circos software (version 0.69) to map the distribution of these genes across chromosomes (refer to Figure 4). Our analysis revealed that the 21 *TrBAMs* were distributed across 16 chromosomes, with a predominant presence on chromosomes TrChr1O, TrChr2O, TrChr2P, TrChr4O, TrChr4P, TrChr5O, TrChr5P, TrChr6O, TrChr7O, and TrChr8P. In contrast, a smaller number of *BAM* genes was observed on TrChr2O, TrChr2P, and TrChr7O, each hosting only one *BAM* gene. Our findings suggest that gene duplication, primarily through segmental duplications (SDs) and some tandem duplications (TDs), contributed to the expansion of the TrBAM gene family. Specifically, six segmental duplications resulted from the amplification of *BAMs* on different chromosomes, while one tandem duplication arose from the generation of *BAM* gene clusters predominantly located on TrChr6O. Some chromosomes, such as TrChr2O and TrChr2P as well as TrChr4O and TrChr4P, displayed similar distributions of *BAMs*, indicating a degree of conservation between homologous chromosomes. Regions containing TD genes appeared to be hotspots of gene distribution, signifying the role of these duplications in the expansion of the TrBAM gene family within the white clover genome.

**Figure 5:**
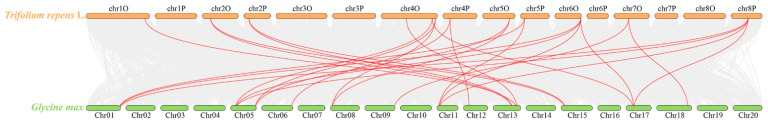
Synteny analyses of *BAMs* between white clover and soybean. The collinear gene pairs with *TrBAM*s are highlighted through the red lines, while the collinear blocks are marked through gray lines.

To unveil the origin and evolution of white clover BAM family members, we conducted a synteny analysis comparing white clover *BAMs* with those of soybean (*Glycine max*) (see Figure 5). The red lines in the background emphasize the syntenic BAM gene pairs present in the genomes of both white clover and soybean, while the gray lines represent the covariate blocks. The identified pairs of syntenic genes exhibited conserved synteny between white clover and soybean. These included 12 *TrBAMs* and 13 soybean genes. All soybean genes contained the “Glyco_hydro_14” domain. This outcome not only underscores the shared evolutionary history of white clover and soybean but also reaffirms their close relationship within the legume family.

Furthermore, we evaluated the selection pressure that acted on *TrBAMs* post-duplication by calculating the ratio of non-synonymous (Ka) to synonymous (Ks) substitutions (Ka/Ks) for duplicate gene pairs (Table S2). The Ka/Ks values for segmentally duplicated *TrBAM* gene pairs ranged from 0.13 to 0.31, with an average of 0.17, implying that these *TrBAMs* were under strong purifying selection during their evolutionary history. This purifying selection pressure suggests that these genes have been conserved due to their essential roles in the plant’s biological processes.

### 2.6. Genetic Regulation Network Analysis of White Clover BAM Genes

**Figure 6:**
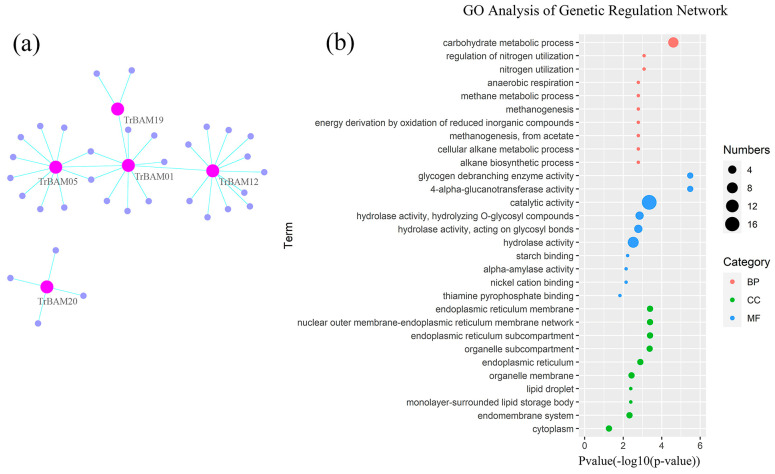
Gene regulatory network and gene ontology enrichment analysis of TrBAMs. (**a**) Gene regulatory network analysis of TrBAMs and their interactions in white clover. The gene regulatory network (GRN) of TrBAMs and their interactions were generated based on Arabidopsis interactions, which were displayed with Cytoscape software (version 3.9.1). Pink nodes correspond to TrBAMs, while violet nodes correspond to the genes that interacted with TrBAMs, the cyan lines represent interactions in white clover; (**b**) gene ontology enrichment analysis of interaction genes with TrBAMs. The GO enrichment analysis showed the involvement of interaction genes with TrBAMs in biological processes, molecular functions, and cellular components. Red dots represent GO terms from a biological process (BP), green dots represent GO terms from molecular function (CC), while blue dots represent GO terms from a cellular component (MF). Dot size represents the number of genes involving the GO term, the *X*-axis represents the *p*-value of topGO enrichment analysis, with −log10 transformation, −log10 (p), while the *Y*-axis represents GO terms.

Gene regulation networks (GRNs) provide valuable insights into the system-level functions of genes. In this study, we reconstructed the GRNs of *TrBAM* genes and their interactions using a public interaction database. The resulting GRNs comprised 38 genes and 38 interactions, as illustrated in Figure 6a. The analysis of these networks revealed that most *TrBAMs* interacted with dozens of functional genes, highlighting their roles in transcriptional regulation processes. For instance, *TrBAM12* was found to interact with 12 genes, *TrBAM05* with 11 genes, and *TrBAM01* with eight genes, underscoring the significance of these *TrBAMs* in the lifespan of white clover. To gain a deeper understanding of the roles of these interacting genes, we retrieved gene ontology (GO) annotations and conducted GO enrichment analysis using the topGO package within the R platform, referred to in Figure 6b. The results indicated that these genes were primarily associated with the organelle membrane, as depicted in Figure 6b, providing supporting evidence that *TrBAMs* are involved in organelle membrane functions. Furthermore, the molecular functions of these genes were primarily linked to catalytic activities, with a strong focus on participating in carbohydrate metabolic processes, thus reinforcing the known functions of *TrBAMs*. This network analysis helps elucidate the broader functional context of *TrBAMs* within the white clover genome.

### 2.7. Expression Analysis of TrBAMs in Response to Cold Stress

**Figure 7:**
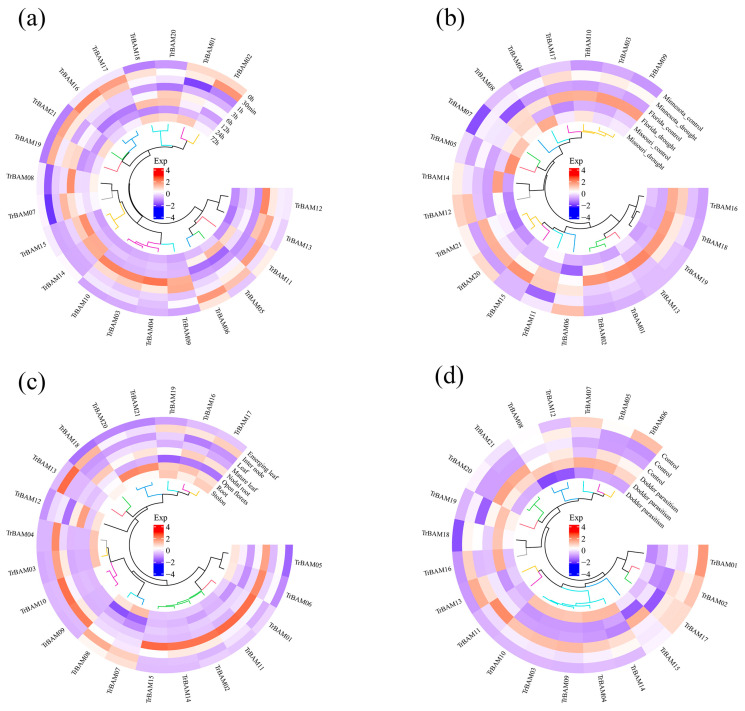
The expressional profiles of TrBAMs under various tissues and stresses. The details of the experiments are as follows: (**a**) Cold stress: RNA-seq database accession numbers of PRJNA781064. Eight time points were examined under cold stress as follows: 0 h, 30 min, 1 h, 3 h, 6 h, 12 h, 24 h, and 72 h, with three biological replications at each time point. (**b**) Drought stress: RNA-seq database accession numbers of PRJNA953427. Three white clover varieties—Minnesota, Florida, and Missouri—were studied under drought stress, with three biological replications for each variety. (**c**) Tissue-specific expression: RNA-seq database accession numbers of PRJNA523044. Eight tissues of white clover were analyzed, including emerging leaf, internode, leaf, mature leaf, nodal root, open florets, root, and stolon. Each tissue type was replicated three times. (**d**) Dodder parasitism: RNA-seq database accession numbers of PRJNA828029. White clover plants were subjected to both control and dodder parasitism conditions, with three biological replications for each group. Mean expression levels (FPKM values) were measured using Salmon software (version 0.12.0) and they were displayed using the ggplots package of the R platform.

To gain a deeper understanding of *TrBAM* involvement in responding to both biotic and abiotic stresses, we examined RNA-seq data collected across various tissues and stresses. White clover plants were exposed to cold stress at 4 °C and RNA-seq analyses were conducted at the eight distinct time points of 0 h, 30 min, 1 h, 3 h, 6 h, 12 h, 24 h, and 72 h. We compiled all *TrBAMs* with an FPKM (fragments per kilobase million) value greater than one; their expression levels were visualized using a violin plot. Figure S2 illustrates that *TrBAMs* exhibited heightened expression in response to cold stress, particularly at 3 h, 6 h, and 12 h, maintaining a high expression level during these stages. These results strongly indicate the crucial roles *TrBAMs* play under cold stress conditions. To further elucidate the expression patterns of *TrBAMs*, we employed hierarchical clustering and heatmap visualization (Figure 7a). Most *TrBAMs* displayed increased expression levels at 3 h, 6 h, and 12 h in response to cold stress, corroborating the findings from the violin plot analysis and reinforcing the responsiveness of these genes to cold stress. Notably, specific *TrBAM* genes, such as *TrBAM03*, *TrBAM04*, *TrBAM09*, *TrBAM10*, *TrBAM12*, *TrBAM13*, *TrBAM14*, and *TrBAM15*, exhibited particularly high expression levels during these critical time points. In Figure 7b, we observed divergent expression patterns of *TrBAMs* in response to drought stress across three varieties of white clover. The *TrBAMs* were significantly up-regulated in Florida under drought stress, with *TrBAM01*, *TrBAM02*, and *TrBAM13* showing significant up-regulation. The tissue-specific expression pattern of *TrBAMs*, depicted in Figure 7c, revealed significantly higher expression levels in leaves compared with other tissues. Finally, in Figure 7d, the up-regulation of *TrBAM04*, *TrBAM09*, and *TrBAM10* in the roots of white clover under dodder parasitism suggests their pivotal role in defending against dodder parasitism. These results underscore the tissue-specific variability in *TrBAM* expression and emphasize their crucial contributions to responding to both biotic and abiotic stresses.

### 2.8. qRT-PCR Validation of TrBAMs Expression in Response to Cold Stress

**Figure 8:**
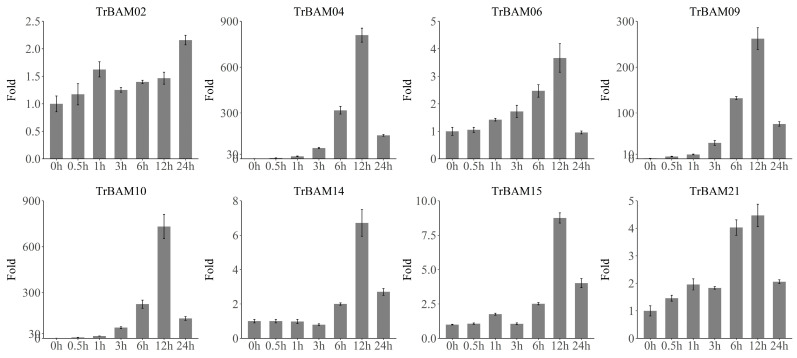
qRT-PCR analysis of TrBAMs in response to cold stress. The *X*-axis represents time points in response to cold stress and the *Y*-axis represents relative expression levels of TrBAM genes, which set the expression level at the “0 h” time point as 1. The expression levels were calculated using the 2^−ΔΔCT^ method as the Section 5.8 describes.

To further validate the significant response of *TrBAMs* to cold stress, we conducted quantitative reverse transcription-polymerase chain reaction (qRT-PCR) analysis of eight selected *TrBAMs* at seven different time points as follows: 0 h, 30 min (0.5 h), 1 h, 3 h, 6 h, 12 h, and 24 h. The qRT-PCR results, as shown in Figure 8, confirmed the substantial upregulation of these *TrBAMs* in response to cold stress. In each case, the genes exhibited a notable increase in expression followed by a gradual decrease. The congruence between the qRT-PCR and RNA-seq results demonstrates a consistent expression pattern for *TrBAMs* in response to cold stress. These findings provide compelling evidence that *TrBAMs* are actively involved in responding to cold stress conditions and play a critical role in regulating gene expression in white clover.

### 2.9. Homology Modeling of BAMs in White Clover

**Figure 9:**
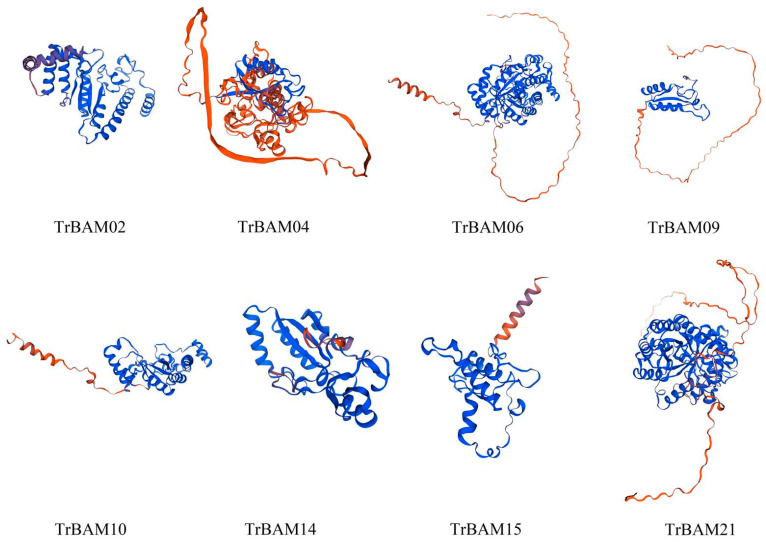
Tertiary structure of eight TrBAM proteins.

The secondary structure predictions of 21 TrBAM proteins revealed that they predominantly consisted of random coils (42.05–47.51%) followed by α-helices (39.22–40.35%), extended strands (12.90–22.78%), and β-turn structures (5.83–13.78%), as summarized in Table 1. Notably, no signal peptides or transmembrane regions were detected in any of the TrBAM proteins. Based on the prediction from SWISS-MODEL (https://swissmodel.expasy.org (accessed on 15 September 2023)), the protein tertiary structures of eight candidate TrBAM proteins were constructed (Figure 9). This structural analysis provides valuable insights into the composition and arrangement of secondary structures within TrBAM proteins, aiding in a deeper understanding of their functional characteristics.

## 3. Discussion

White clover is a nutrient-rich perennial leguminous forage. The sequencing of the white clover genome has enabled us to identify stress-resistant genes and promote genetic improvement. Since BAM has been shown to respond to stress, it is essential to study the functionality and mechanisms of the TrBAM gene family [37,38].

BAM is a multi-gene family found in several different plant species. *Arabidopsis thaliana* has nine *BAM* genes in its genome, rice has four *BAM* genes, and barley has nine *BAM* genes [39,40,41]. In horticultural crops, BAM has also been identified in some species such as 29 *BAM* genes in the banana genome and 19 *BAM* genes in grapes [42,43]. In this study, using bioinformatic methods, 21 members of the TrBAM gene family were identified in white clover. Compared with Arabidopsis (nine members), the BAM gene family in white clover has expanded [17,44]. By analyzing the evolutionary relationship between *TrBAMs* and *AtBAMs*, we found that white clover is closely related to Arabidopsis, which is consistent with the fact that both white clover and Arabidopsis are dicotyledonous plants.

Most studies investigating the function of β-amylase in vivo have primarily focused on the model plant *Arabidopsis thaliana*. The Arabidopsis genome harbors nine BAM isoforms (refer to Table 1). Notably, four of these isoforms (*AtBAM1* to *AtBAM4*) exhibit a propensity for targeting chloroplasts [13,45]. Two other isoforms, namely *AtBAM7* and *AtBAM8*, function as nuclear proteins [14], while *AtBAM5* is identified as a cytoplasmic protein primarily found in sieve elements of the phloem [46,47]. We have predicted subcellular localization of TrBAM proteins and the results have also showed they was mainly located in the nucleus or chloroplasts, which was consisted with the findings in Arabidopsis. These results have also indicated BAM genes were highly conservative in plants and their functions may also be similar across plants. In addition, two *BAM* genes from *Annona atemoya*, *AaBAM3*, and *AaBAM9* also reported localization in chloroplasts [48], while another *BAM* gene from *upland cotton*, *GhBAM7*, was explored as having important roles in fiber development; the GhBAM7 protein was located in the nuclear using GFP localization [49]. *PbBAM3* was cloned from a pear, which was also validated as a chloroplast-localized protein, and its function was well documented [50]. Chloroplasts serve as the primary site for starch catabolism, producing maltose, which is subsequently transported to the cytoplasm. This maltose is intricately involved in sugar metabolism, ultimately contributing to the synthesis of various soluble sugars through a series of enzyme-catalyzed reactions [50].

Gene duplication includes tandem duplication, segmental duplication, and whole-genome duplication. During the course of evolution, gene duplication plays a crucial role in chromosome rearrangement, amplification, functional diversification, and the expansion of gene families [51,52,53]. Recently, Thalmann et al. discovered that gene duplication promoted the extensive expansion and functional diversification of the BAM gene family [54]. To explore whether the TrBAM gene family expanded during evolution, we studied tandem and segmental duplications as these two features are considered important in the expansion of gene families [51,55]. In this study, a total of seven duplication events were identified, including six segmental duplications and one tandem duplication. In general, a Ka/Ks value of < 1 indicates that genes have been influenced by purifying selection during evolution; Ka/Ks = 1 represents neutral selection; and Ka/Ks > 1 indicates positive selection. Since the Ka/Ks values for all pairs of *TrBAM* genes were <1, it is suggested that the TrBAM gene family underwent purifying selection during evolution (Table S2). Therefore, gene duplication is the primary mechanism for the expansion and evolution of the white clover BAM gene family.

This study constructed a gene regulatory network containing 38 interacting genes. Some *TrBAMs* had numerous interacting genes, such as *TrBAM12* that interacted with 12 genes, *TrBAM05* that interacted with 11 genes, and *TrBAM01* that interacted with eight genes. Starch degradation hydrolysis pathways typically include AMY, SS, and BAM. AMY and SS are key carbohydrate hydrolases that catalyze starch degradation [27,56,57]. Gene regulatory network analysis revealed that *TrBAM* genes closely interacted with AMY and DPE. Alpha-amylase (AMY1/3) is a crucial carbohydrate hydrolase that effectively degrades starch when acting in concert with beta-amylase [58]. Meanwhile, 4-alpha-glucanotransferase (DPE1/2) is a cytoplasmic protein with both glucanotransferase and amylase activities, playing an important role in starch-to-sucrose conversion and cellular metabolism [56]. Results from the gene regulation networks suggest that *TrBAM* genes interact with these starch degradation enzymes, jointly regulating starch metabolism. Furthermore, from the GO enrichment analysis, it is evident that the molecular functions of these functional genes are highly concentrated in catalytic activity and are primarily involved in carbohydrate metabolic processes, confirming the function of *TrBAMs*.

The metabolism of sugar is essential for the growth and development of plants. In response to biotic and abiotic stresses, plants regulate carbohydrate conversion [59,60]. Starch serves as an intermediary form of carbon assimilation during fleshy fruit development. Its degradation is closely associated with fruit ripening and metabolism [61,62]. Previous studies have shown that temperature stress can alter *BAM* transcription levels and activity [63], particularly inducing *BAM* production under low-temperature conditions. *BAM* genes regulate starch degradation, participate in plant sugar metabolism, and respond to biotic and abiotic stresses [64]. Our previous RNA-seq analysis illuminated a significant upregulation of *TrBAMs* under cold stress. Figure S2 illustrates that, with the exception of the 72 h sample, the expression levels in all cold-treated samples surpassed those in the control samples. This pattern suggests a notable upregulation of *TrBAMs* under cold stress, particularly during the night, exemplified by *TrBAM04*, *TrBAM09*, and *TrBAM10*. The consistency of these findings with our qRT-PCR results reinforces the reliability of the RNA-seq results. Further exploration of *TrBAM* expression across eight tissues of white clover revealed the highest expression levels in leaves. Given the direct relationship between starch catabolism and leaves, this aligns seamlessly with the expected function of BAMs. RNA-seq analysis unveiled an upregulation of several *TrBAMs* in response to the stress of dodder parasitism, underscoring the active role *TrBAMs* play in responding to biotic stress. Analysis of RNA-seq from white clover subjected to drought stress unveiled positive responses from certain *TrBAMs*, including *TrBAM01*, *TrBAM02*, *TrBAM09*, *TrBAM10*, and *TrBAM13*. This aligns with the study by Prach et al. that reported that *AtBAM1*-regulated starch degradation in guard cells attenuated the stomatal opening in drought-stressed Arabidopsis. Notably, the impaired starch catabolism in *bam1* mutant plants was accompanied by a reduction in stomatal openings [23]. This reinforces the potential role of *TrBAMs* in modulating starch-related processes during drought stress, drawing parallels with similar findings in Arabidopsis. Through multiple sequence alignment of these genes significantly upregulated under various tissues and stresses, we found that *TrBAM04*, *TrBAM09*, and *TrBAM10* are homologous genes to Arabidopsis *AtBAM3*. Previous research has shown that *BAM3* plays a role in degrading leaf starch in mesophyll cells during the night and under low-temperature stress [8,65]. Seki et al. identified a *BAM*-related EST during the screening of cold-induced genes, which was confirmed to be *AtBAM3* [66]. The specific expression of *AtBAM3* is closely related to maltose accumulation, suggesting its pivotal role in leaf starch degradation [66]. Kaplan and Guy also found that *AtBAM3* was significantly induced by low temperatures, with increased expression after exposure to 4 °C [65]. These findings suggest that the expression of *AtBAM3* and its cold resistance capabilities are closely related. This is consistent with our research results. Additionally, we selected the 2,000 bp regions upstream of the *TrBAM* gene loci as promoter regions (refer to Figure 3). Among the 20 identified *cis*-acting elements, we observed DRE binding sites specifically recognized by *CBF* genes in the promoter regions of certain genes, including *TrBAM04*, *TrBAM10*, *TrBAM14*, and *TrBAM16*. Over the past few decades, the *CBF* regulatory pathway has emerged as the most comprehensively understood cold-responsive network [67]. *TrBAM04* and *TrBAM10,* as homologs of *AtBAM3*, have been identified. Given earlier research indicating that *BAM* expression is regulated by *CBF*, this suggests the potential activation of the CBF-BAM module under cold stress, contributing to enhanced cold tolerance [26]. Consequently, further exploration and validation of the molecular regulatory mechanism of *TrBAMs* in response to abiotic stress are warranted in future studies. However, the precise response mechanism of *AtBAM3* under cold stress remains unclear and necessitates more in-depth exploration of its function.

## 4. Conclusions

This study presents a comprehensive analysis of the TrBAM gene family in white clover. Twenty-one *TrBAMs* were identified and classified into four groups. Their conserved motifs, *cis*-acting elements, chromosome localization, gene duplication events, synteny analysis, protein interaction networks, and tertiary structural models were analyzed. We found that the evolution and expansion of the BAM gene family may be closely related to segmental and tandem duplications within *BAMs*. Gene regulatory network analysis revealed that *TrBAMs* have close interactions with AMY and DPE. Additionally, our findings suggest that *BAMs* might be involved in various tissues and stresses. The expression profiles of *TrBAMs* in response to biotic and abiotic stresses were analyzed using RNA-seq data, confirming their regulatory roles in biotic and abiotic stresses responses. The qRT-PCR results further demonstrated the significant role of *BAMs* in white clover’s response to cold stress. This study provides a foundation for further research on the function of the BAM gene family in white clover under cold stress.

## 5. Materials and Methods

### 5.1. Identification and Classification of the TrBAM Gene Family in White Clover

White clover genome resource information was obtained from a prior study and all data files were graciously provided by Stig Uggerhøj Andersen of Aarhus University [35]. DNA, CDS, and protein sequences were extracted from the white clover genome. We acquired the accession numbers for the nine *BAM* genes of *Arabidopsis thaliana*, referring to the research conducted by Fulton et al., and subsequently downloaded their complete coding sequences (CDSs) [13]. These nine Arabidopsis BAM protein sequences were employed as query sequences for a BLAST search (version 2.9.0+) of the white clover genome using an E-value threshold of 1E-05 and a minimum coverage of 80% [68]. Subsequently, a hidden Markov model (HMM) profile (version 3.3) (PF01373) was retrieved by searching the Pfam database [69]. To identify and validate BAM gene family members in the white clover genome, HMMER3.1 was utilized, designating them as candidate BAM proteins [70]. An expect (e) cutoff of 0.01 was employed. We identified and named 21 TrBAM gene family members, denoting them as *TrBAM1* to *TrBAM21*. Comprehensive annotations for all candidate *BAM* genes were extracted from the white clover genome, including information about their genomic positions, protein lengths, and intron numbers. To predict their protein secondary structures, we employed the SOPMA tool (https://npsa-prabi.ibcp.fr/cgi-bin/npsa_automat.pl?page=npsa%20_sopma.html) (accessed on 12 October 2023). Finally, all white clover *BAM* gene members were categorized into groups based on their similarity to *BAM* genes in Arabidopsis. The WoLF PSORT predictor was used to predict the subcellular localization of TrBAM proteins (https://wolfpsort.hgc.jp) (accessed on 23 November 2023).

### 5.2. Phylogenetic Analysis of the TrBAM Genes in White Clover

The candidate white clover BAM protein sequences were subjected to multiple sequence alignment using MUSCLE (version 5.1.0) with default parameters [71]. A phylogenetic tree was constructed using the protein sequences from Arabidopsis via MEGA 11, utilizing the Neighbor-Joining (NJ) method, and supported by 1,000 bootstrap replicates [72]. The *TrBAMs* were classified into distinct groups based on their placement in the phylogenetic tree relative to AtBAM and TrBAM protein sequences.

### 5.3. Motif Composition Distribution Analysis of TrBAMs in White Clover

Conserved motif structures within the TrBAM protein sequences were determined using the MEME Suite (multiple EM for motif elicitation, version 4.8.1) with specific parameters as follows: zero or one occurrence per sequence of site distribution, a maximum of 10 misfits, and a maximum motif width ranging from 6 to 50 [73]. All results were visualized using TBtools (version 1.098) [74].

### 5.4. Cis-Acting Element Analysis in the Promoters of TrBAM Gene Family Members

The genomic loci of the *TrBAM* genes and the 2000 bp regions upstream of these loci were selected as promoter regions. These regions were analyzed for *cis*-acting elements using the PlantCARE online tool (http://bioinformatics.psb.ugent.be/webtools/plantcare/html/) (accessed on 21 November 2023).

### 5.5. Chromosomal Location, Gene Duplication, and Ka/Ks Analysis of TrBAM Genes in White Clover

All white clover proteins were compared to each other using BLASTP software (version 2.9.0+). Subsequently, gene duplications were identified and characterized based on the BLAST results using MCScanX (version python) [75]. To explore the syntenic relationships of the *BAMs* obtained from white clover and soybean (*Glycine max*), syntenic analysis maps were constructed using MCScanX [75]. The CIRCOS software (version 0.69) was employed to visualize the distribution of the BAM gene family in the white clover genome, considering the positional information of *BAMs* within the white clover genome and the occurrence of gene duplications [76]. To assess selection pressure on specific genes following duplication events, Ka/Ks ratios were determined. Ka/Ks ratios of = 1, >1, and < 1 indicated neutral, positive, and purifying selection, respectively [77].

### 5.6. Gene Regulation Network Analysis of White Clover TrBAM Gene Family

Information regarding the gene regulatory network (GRN) of Arabidopsis was sourced from the AraNet database (V2) [78], encompassing 22,894 Arabidopsis genes and 895,000 interactions (links). To establish homologous relationships, all proteins from white clover and Arabidopsis underwent reciprocal BLAST searches. These searches were performed with an E-value threshold of 1 × 10^−5^ and the highest-scoring hits were identified as either homologs of white clover genes or homologs of Arabidopsis genes. This process produced pairs of homologous genes based on two BLAST results. The Arabidopsis GRN was then employed to construct the white clover GRN using information from these homologous gene pairs. Subnetworks containing the white clover *TrBAM* genes were sought and analyzed, with the results visualized using Cytoscape software (version 3.9.1) [79]. Gene ontology (GO) enrichment analysis was carried out on these subnetworks using topGO (version 2.38.31), applying a significance threshold of 0.05 [80]. The results provide an overview of the most crucial terms and identify the highly enriched terms as functions within the GRN, following the software’s protocol.

### 5.7. Expression Analysis of White Clover TrBAMs in Response to Cold Stress

The RNA-seq datasets, encompassing responses to cold stress at eight time points, three white clover varieties under drought stress, eight distinct tissues, and dodder parasitism, can be accessed using the accession numbers as follows: PRJNA781064, PRJNA953427, PRJNA523044, and PRJNA828029, respectively [35,81,82]. These data were leveraged to investigate the expression patterns of *TrBAM*. The Salmon software (version 0.12.0) was used to align the RNA-seq readings to the transcript sequences of the white clover genome; the expression levels (FPKM values) of each gene were calculated using Salmon [83]. To standardize the expression data, a logarithmic transformation was applied (log2), and centering was performed using the “scale” function of the R program (version 4.2.1). Subsequently, the expression data were clustered and visualized using the “heatmap.2” function of the ggplots package (version 3.1.3).

### 5.8. Plant Growth and qRT-PCR Analysis

Seeds of white clover cv. Haifa were procured from Barenbrug China Ltd., Com. (Beijing, China). These seeds were germinated and transplanted into a mixture of perlite and sand at a 3:1 volume ratio, following our previously outlined protocol [81]. In summary, the seeds were sown in pots, with approximately 10–15 plants per pot. They were then placed in an environmental growth chamber set at a light intensity of 100 µmol/(m·s) at 24/18 °C (day/night), with a 12 h photoperiod. The plants were irrigated with half-strength Hoagland solution every two days. After four weeks, they were randomly divided into seven groups for cold stress treatment, collecting samples at 0 h (control), 30 min, 1 h, 3 h, 6 h, 12 h, and 24 h (a total of seven time points) at a temperature of 4 °C. Cold stress was initiated at 8:00 a.m. and lasted until 8:00 a.m. the following morning. For each group, three samples were randomly chosen and five seedlings were pooled to constitute a biological replicate. All samples were flash-frozen in liquid nitrogen and stored at −80 °C.

Total RNA extraction was performed using the RNApure Plant Kit (Tiangen, Beijing, China). Subsequently, cDNA synthesis was conducted using the Prime Script RT kit (Toyobo, Shanghai, China) to serve as a template for the quantitative reverse transcription-PCR (qRT-PCR). Primers were designed using Primer3 based on the nucleotide sequences of BAM family genes (Table S3) [84]. RT-qPCR analysis was conducted with three replicates of each experiment. The RT-qPCR conditions and reaction systems followed the protocol for SYBR Premix Ex TaqTMII (Toyobo, Shanghai, China). Data acquisition was executed using the LightCycler^®^ 96 system (Roche, Basel, Switzerland). Fold change values were calculated based on gene expression abundance, employing the 2^−ΔΔCT^ method [85,86].

### 5.9. Homology Modeling of BAMs in White Clover

Homology modeling was employed to predict the 3D structure of the TrBAM gene family members. Protein tertiary structures were modeled using the SWISS-MODEL homology modeling server (https://www.string-db.org/) (accessed on 12 October 2023). The intensive modeling mode was selected for this analysis. Model quality was evaluated using the SAVES server (https://saves.mbi.ucla.edu/) (accessed on 12 October 2023).

## Figures and Tables

**Table 1:** Summary of *TrBAM* genes identified in the white clover.

Name	Locus	Chromosomal Locations	Arabidopsis Homologous Gene		Intron	Length (aa)	Secondary Structure			
			Gene Accession NO.	Gene Name			Alpha Helix (%)	Random Coil (%)	Extended Strand (%)	Beta Turn (%)
*TrBAM01*	chr6.jg1386	Tr6O:9493308 -9497964	AT3G 23920	*BAM1*	3	566	39.22	42.05	12.9	5.83
*TrBAM02*	chr6.jg2160	Tr6O:1496895 -14973417	AT3G 23920	*BAM1*	3	566	37.28	36.40	16.08	10.25
*TrBAM03*	chr13.jg887	Tr5P:5728535 -5730683	AT4G 15090	*BAM3*	3	358	35.75	46.93	11.45	8.87
*TrBAM04*	chr13.jg890	Tr5P:5734041 -5735464	AT4G 15090	*BAM3*	2	294	28.23	45.92	18.03	7.82
*TrBAM05*	chr16.jg3573	Tr8P:25387801 -25390377	AT4G 15090	*BAM3*	3	545	33.76	44.77	15.78	5.69
*TrBAM06*	chr4.jg10937	Tr4O:7689286 -76895578	AT4G 15090	*BAM3*	3	545	35.05	44.22	14.86	5.87
*TrBAM07*	chr4.jg11652	Tr4O:8180919 -81812477	AT4G 15090	*BAM3*	3	553	34.18	38.70	16.46	10.67
*TrBAM08*	chr5.jg6019	Tr5O:4042454 -40428441	AT4G 15090	*BAM3*	4	583	31.39	47.51	14.75	6.35
*TrBAM09*	chr6.jg5766	Tr6O:3908281 -39084765	AT4G 15090	*BAM3*	3	368	28.53	39.67	20.65	11.14
*TrBAM10*	chr6.jg5768	Tr6O:3909020 -39092831	AT4G 15090	*BAM3*	3	531	34.46	39.92	17.14	8.47
*TrBAM11*	chr1.jg4857	Tr1O:3462597 -34628010	AT4G 15210	*BAM5*	6	225	37.33	29.78	19.11	13.78
*TrBAM12*	chr12.jg1584	Tr4P:10584000 -10586714	AT4G 15210	*BAM5*	6	496	33.47	33.67	22.04	10.82
*TrBAM13*	chr12.jg4565	Tr4P:29991828 -29994397	AT4G 15210	*BAM5*	6	496	33.06	34.68	22.78	9.48
*TrBAM14*	chr1.jg12949	Tr1O:8919646 -89201011	AT2G 32290	*BAM6*	7	590	31.53	37.46	21.02	10.00
*TrBAM15*	chr4.jg5171	Tr4O:3786960 -37870869	AT2G 32290	*BAM6*	2	171	40.35	46.78	6.43	6.43
*TrBAM16*	chr16.jg2829	Tr8P:20373202 -20379501	AT2G 45880	*BAM7*	9	700	31.71	38.29	19.86	10.14
*TrBAM17*	chr7.jg3516	Tr7O:2201647 -22022486	AT2G 45880	*BAM7*	9	700	33.57	36.86	19.00	10.57
*TrBAM18*	chr1.jg6953	Tr1O:4886182 -48866567	AT5G 45300	*BAM8*	6	389	37.02	29.05	22.37	11.57
*TrBAM19*	chr5.jg1632	Tr5O:1149921 -11502257	AT5G 45300	*BAM8*	4	335	31.34	46.57	15.22	6.87
*TrBAM20*	chr10.jg1089	Tr2P:7394929 -7397652	AT5G 18670	*BAM9*	2	534	34.83	46.25	14.04	4.87
*TrBAM21*	chr2.jg1872	Tr2O:1276248 -12765223	AT5G 18670	*BAM9*	2	534	34.27	45.69	15.54	4.49

## Data Availability

The datasets presented in this study can be found in online repositories. The names of the repository/repositories and accession number(s) can be found in the article/Supplementary Material.

## Supplementary Materials

The following supporting information can be downloaded at: https://www.mdpi.com/article/10.3390/plants13020154/s1; Figure S1: sequence logos for motifs identified from *TrBAM* genes; Figure S2: the violin plot of all *TrBAM* genes based their expression levels in response to cold stress; Table S1: subcellular localization of TrBAMs in the white clover; Table S2: Ka/Ks values of TrBAM gene pairs in the white clover; Table S3: primers used for qRT-PCR analysis of the TrBAMs.
