# Supplementary material for: Comprehensive Identification of the β-Amylase (BAM) Gene Family in Response to Cold Stress in White Clover
Source: Plants (Basel). 2024 Jan 5;13(2):154. doi: 10.3390/plants13020154 (PMC10820397; doi:10.3390/plants13020154)
Supplement: Supplementary file 1 [file plants-13-00154-s001.zip › Table S1.pdf]

Table S1 Subcellular Localization of TrBAMs in the white clover

Name	Subcellular Localization
TrBAM01	chlo: 4, extr: 4, cyto: 2, cysk_plas: 1.33333, mito_plas: 1.33333, nucl: 1, E.R.: 1
TrBAM02	nucl: 4, cysk: 4, cyto: 3, chlo: 2, plas: 1
TrBAM03	nucl: 7, mito: 4, cyto: 2, extr: 1
TrBAM04	cysk: 6, nucl: 4, cyto: 2, extr: 2
TrBAM05	mito: 6, nucl: 3, cyto: 3, chlo: 2
TrBAM06	mito: 5, chlo: 3, nucl: 3, cyto: 3
TrBAM07	nucl: 5, chlo: 4, cyto: 1, mito: 1, plas: 1, vacu: 1, golg: 1
TrBAM08	chlo: 13, nucl: 1
TrBAM09	cyto: 6, cysk: 5, nucl: 2, chlo: 1
TrBAM10	chlo: 5, mito: 2, E.R.: 2, cyto: 1, plas: 1, extr: 1, vacu: 1, cysk_nucl: 1
TrBAM11	cyto: 9, nucl: 2, mito: 2, chlo: 1
TrBAM12	extr: 4, chlo: 3, nucl: 2, E.R.: 2, cysk_plas: 1.33333, mito_plas: 1.33333, vacu: 1
TrBAM13	cyto: 7, nucl: 5, mito: 1, pero: 1
TrBAM14	cyto: 8, nucl: 6
TrBAM15	chlo: 4, nucl: 4, plas: 2, cysk: 2, cyto: 1, extr: 1
TrBAM16	nucl: 8, chlo: 4, cyto: 1, cysk: 1
TrBAM17	nucl: 8, chlo: 4, cyto: 1, cysk: 1
TrBAM18	nucl: 11, chlo: 2, cyto: 1
TrBAM19	nucl: 6.5, cyto_nucl: 6, cyto: 4.5, cysk: 2, chlo: 1
TrBAM20	nucl: 10, cyto: 2, vacu: 1, cysk: 1
TrBAM21	nucl: 8, chlo: 2, cyto: 2, vacu: 1, cysk: 1
