# Supplementary material for: Comprehensive Identification of the β-Amylase (BAM) Gene Family in Response to Cold Stress in White Clover
Source: Plants (Basel). 2024 Jan 5;13(2):154. doi: 10.3390/plants13020154 (PMC10820397; doi:10.3390/plants13020154)
Supplement: Supplementary file 1 [file plants-13-00154-s001.zip › Table S2 .pdf]

Table S2 Ka/Ks values of *TrBAM* gene pairs in the white clover

Gene_1	Gene_1	Ka	Ks	Ka/Ks
TrBAM14	*TrBAM15*	0.010008489	0.076014371	0.131665746
TrBAM21	*TrBAM20*	0.009781418	0.111369477	0.087828538
TrBAM07	*TrBAM08*	0.003962498	0.012706784	0.311841107
TrBAM06	*TrBAM05*	0.000789214	0.033131681	0.023820528
TrBAM09	*TrBAM05*	0.162349738	0.880713557	0.184338865
TrBAM09	*TrBAM10*	0.053795759	0.203640137	0.264170709
