# Supplementary material for: Comprehensive Identification of the β-Amylase (BAM) Gene Family in Response to Cold Stress in White Clover
Source: Plants (Basel). 2024 Jan 5;13(2):154. doi: 10.3390/plants13020154 (PMC10820397; doi:10.3390/plants13020154)
Supplement: Supplementary file 1 [file plants-13-00154-s001.zip › Table S3.pdf]

Table S3 Primers used for qRT-PCR analysis of the *TrBAM*s

Name	Forward primer sequences (5’→3’)	Reverse primer sequences (5’→3’)
TrBAM02	TGGTACTCTCAGATGCTGTT	AACATTTGAGCAATCGGGAG
TrBAM04	TTTCTTGTCCAAAAATCAAACCA	CAAATACCGGTACTCTAGTCG
TrBAM06	TTCTATGCAAGTAGCACACAC	AAACTAGCACTCATCGCTCT
TrBAM09	AATCTCTTGATGATGTTTTTGCC	GGTTATGCGCAACCGAT
TrBAM10	TTTCTTGTCCAAAAATCAAACCA	CAAATACCGGTACTCTAGTCG
TrBAM14	CTATACGTGTGGAAAGAGCAA	GATTTTCGTACGTTGGAGGAA
TrBAM15	TGGTGTCACATACCTACGTT	TCAGACCATGGATATGGCTT
TrBAM21	ACAGGGAGATGGTGTAAGAT	TTTCCCATGGCTTCTTTCTC
Action	TGCTTGATTCCGGTGATGGTGTG	TTCTCGGCAGAGGTACTGAAGGAG
GAPDH	TTTTTCAGCCATGGGCAA	TCATCGTTTTTCCACTGTCC
