# Supplementary figures and images for: Comprehensive Identification of the β-Amylase (BAM) Gene Family in Response to Cold Stress in White Clover
Source: Plants (Basel). 2024 Jan 5;13(2):154. doi: 10.3390/plants13020154 (PMC10820397; doi:10.3390/plants13020154)

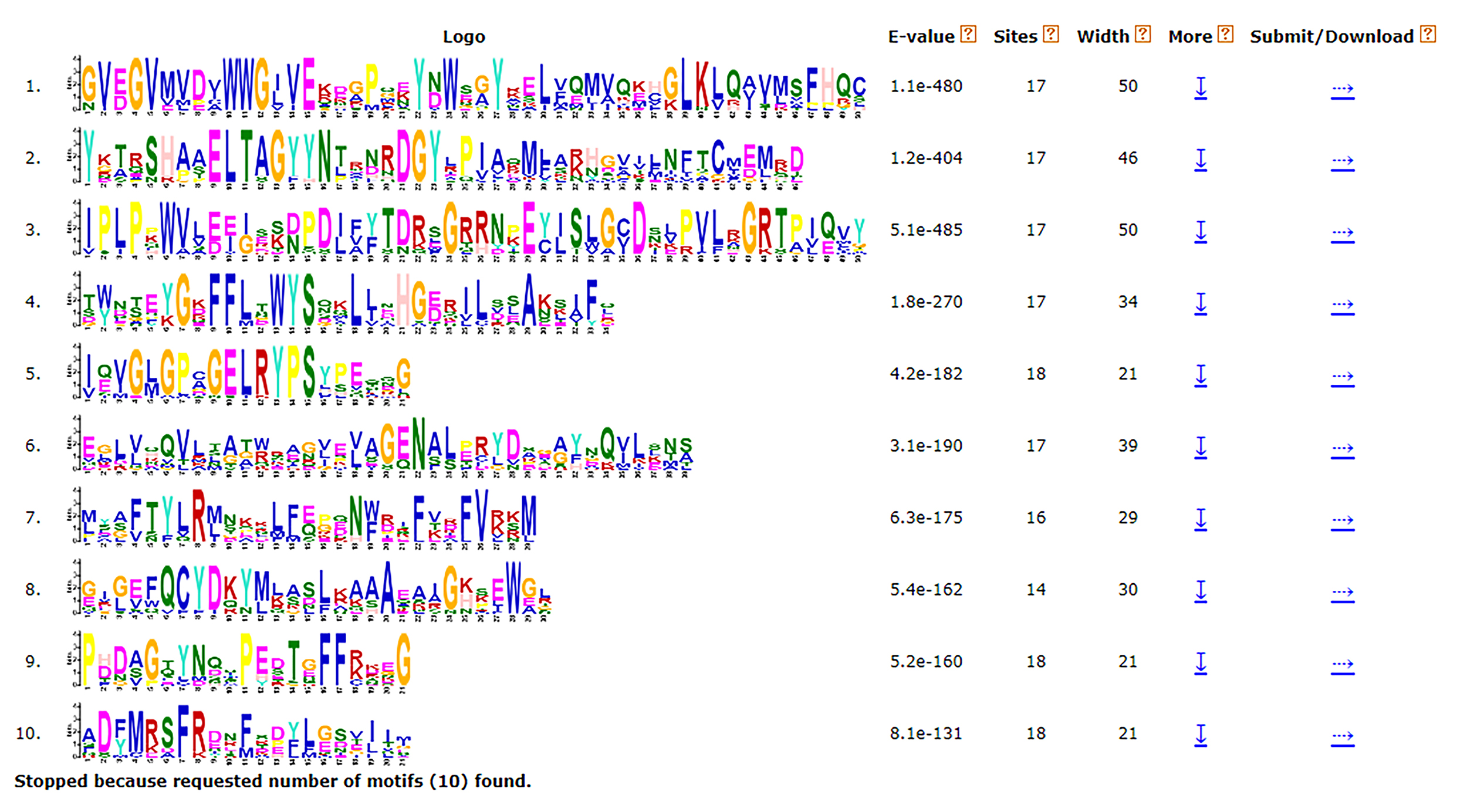

Supplement: Supplementary file 1 [file plants-13-00154-s001.zip › Figure S1 Sequence logos.jpg]

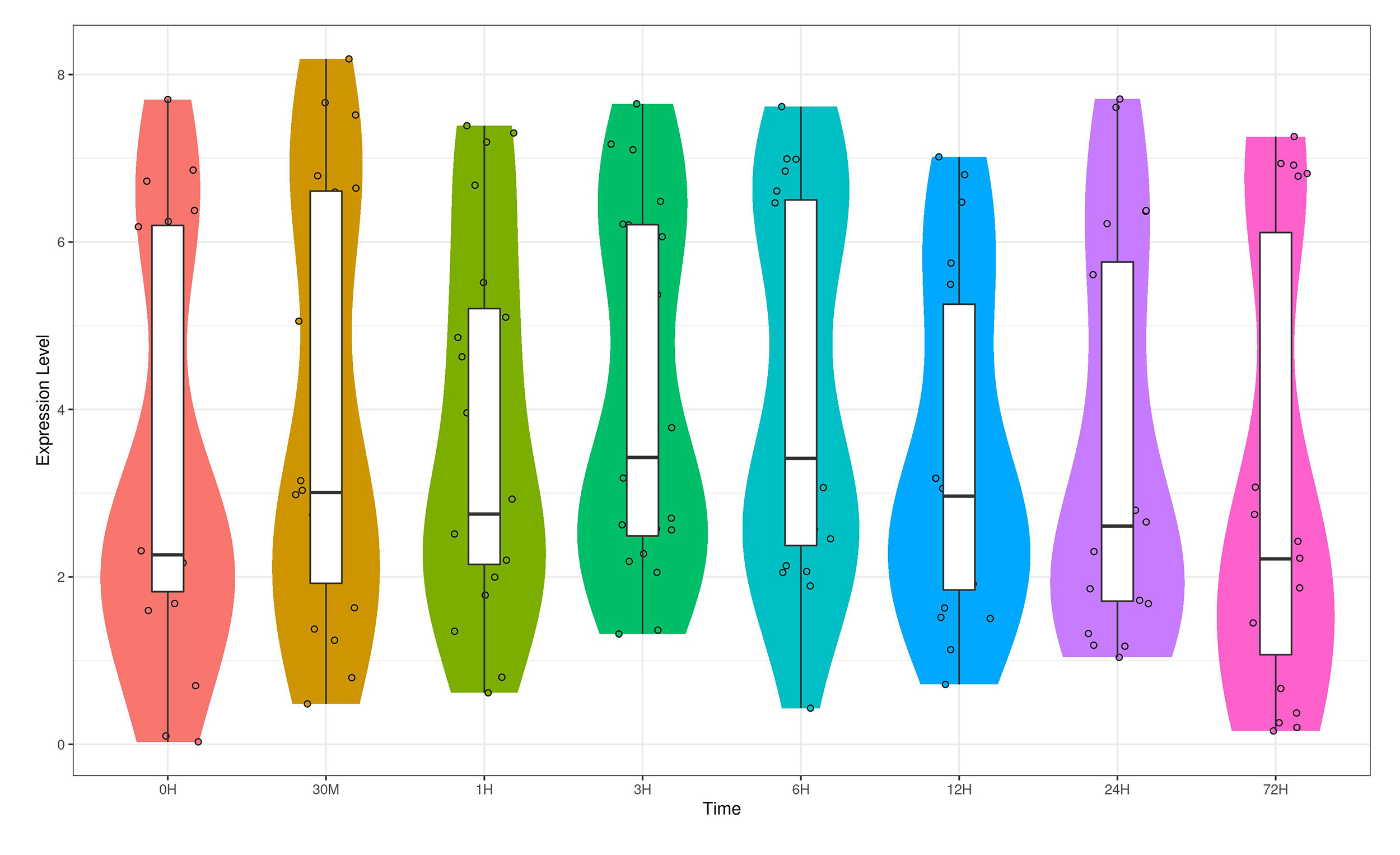

Supplement: Supplementary file 1 [file plants-13-00154-s001.zip › Figure S2 Violin plot.jpg]
